# Advances in Understanding Vector Behavioural Traits after Infection

**DOI:** 10.3390/pathogens10111376

**Published:** 2021-10-24

**Authors:** Nouman Javed, Asim Bhatti, Prasad N. Paradkar

**Affiliations:** 1 CSIRO Health & Biosecurity, Australian Centre for Diseases Preparedness, Geelong, VIC 3220, Australia; njaved@deakin.edu.au; 2 Institute for Intelligent Systems Research and Innovation (IISRI), Deakin University, Geelong, VIC 3220, Australia; asim.bhatti@deakin.edu.au

**Keywords:** vector-borne diseases, vector behaviour, mosquito, ticks

## Abstract

Vector behavioural traits, such as fitness, host-seeking, and host-feeding, are key determinants of vectorial capacity, pathogen transmission, and epidemiology of the vector-borne disease. Several studies have shown that infection with pathogens can alter these behavioural traits of the arthropod vector. Here, we review relevant publications to assess how pathogens modulate the behaviour of mosquitoes and ticks, major vectors for human diseases. The research has shown that infection with pathogens alter the mosquito’s flight activity, mating, fecundity, host-seeking, blood-feeding, and adaptations to insecticide bed nets, and similarly modify the tick’s locomotion, questing heights, vertical and horizontal walks, tendency to overcome obstacles, and host-seeking ability. Although some of these behavioural changes may theoretically increase transmission potential of the pathogens, their effect on the disease epidemiology remains to be verified. This study will not only help in understanding virus–vector interactions but will also benefit in establishing role of these behavioural changes in improved epidemiological models and in devising new vector management strategies.

## 1. Introduction

Vector-borne diseases are caused by bacteria, viruses, or parasites and are spread by vectors between susceptible hosts including wildlife, domestic animals, and humans. According to the World Health Organisation (WHO), annually over 700,000 deaths are caused by vector-borne diseases, including malaria, dengue, yellow fever, and Japanese encephalitis [1]. Mosquito-borne diseases are the deadliest diseases among all vector-borne diseases (World Health Organisation, 2015), while tick-borne diseases are the most rapidly spreading diseases (World Health Organisation, 2017). Malaria causes an estimated 220 million cases globally and leads to more than 400,000 deaths yearly, with children under the age of 5 years being the most vulnerable group [2,3]. For viral infections, dengue is the most prevalent, with an estimated 390 million infections worldwide and around 20,000 deaths per annum [4]. Other vector-borne diseases such as leishmaniasis, schistosomiasis, and Chagas disease also infect hundreds of millions of people worldwide [1], causing high morbidity and mortality.

Studies of the interactions between vectors and their pathogens are vital to understanding vector-borne disease transmission and epidemiology. These can include cellular interactions leading to molecular changes in the vector [5,6] or interactions leading to changes in pathogen transmission [7]. For an arthropod, being a competent vector means being capable of attaining, sustaining, and transmitting a pathogen, such as a virus [8]. For an infectious agent, being proficient requires it to be transmitted to susceptible hosts [9]. Although vector-borne pathogens cause disease in vertebrate hosts during their life cycles, there is no overt disease in vectors. This may be due to the adaptation and long co-evolution of vectors and pathogens.

Infections with many pathogens can modulate different behavioural traits of their arthropod hosts [10,11,12]. However, whether these behavioural changes due to infection are a result of direct manipulation by the infectious agent leading to its transmission advantage is not determined for the for the numerous vector and pathogen species recorded. Similarly, few studies have documented whether infectious agents alter the fitness of the arthropod host by manipulating its behaviour. For instance, increased biting rate that increases the infectious agent’s transmission probability may not improve the vector’s fitness. The propensity of a vector to bite competent or noncompetent hosts is vital for infectious agents’ transmission. Epidemiological models have revealed that arthropod vectors’ preference for infected hosts can enhance spread during the initial stage of an epidemic or outbreak [13,14]. On the other hand, extreme preference for both infected and uninfected hosts can also restrict disease spread [15].

Despite more than a century of research, vector-borne diseases still pose a huge burden on public health worldwide [4,16,17]. It is important to understand the effect of infection on vector behavioural traits to understand its impact on the transmission and epidemiology of the disease. This review paper assesses how infection with pathogens can modulate vectors behaviour, focusing on mosquitoes and ticks (Table S1).

## 2. Behavioural Changes in Mosquitoes after Infection

### 2.1. Malaria and Behavioural Modulations in Mosquitoes

Malaria is a mosquito-borne disease caused by the *Plasmodium* parasite which has several species that can infect different animal hosts [18]. *Plasmodium* lifecycle includes several stages in the two hosts, which includes *Anopheline* mosquito. When a mosquito bites the infected person, it takes the gametocytes along with the blood. The gametocyte then matures into the macrogamete (female) and microgametes (male). In the mosquito gut, the fertilisation of microgamete with macrogamete lead to formation of zygotes. The zygote turns into an ookinete, which penetrates the midgut wall and forms oocysts. Inside the oocyst, the nucleus of the ookinete divides into several thousand sporozoites. The sporozoites enter into the mosquito haemolymph to reach the salivary glands. During the next feeding, these sporozoites are injected into the host blood to continue the lifecycle with specific developmental stages in human.

#### 2.1.1. Changes in Fitness

**Figure 1:**
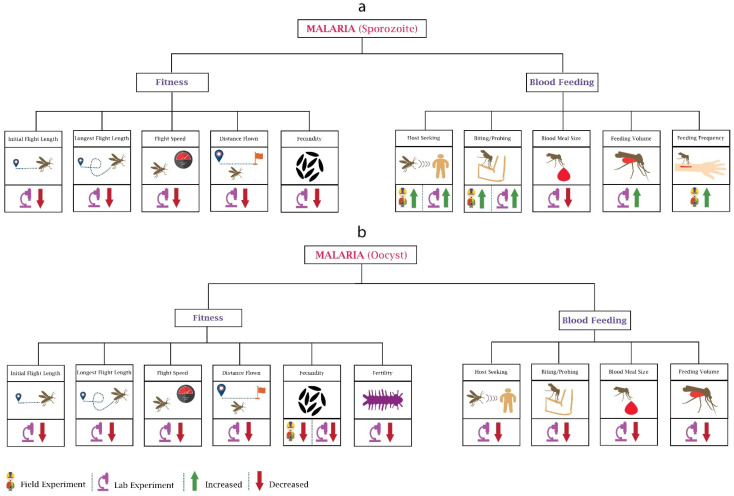
Behavioural changes in mosquitoes due to malaria parasite: The major two stages of the malaria parasite (*Plasmodium*) are sporozoite and oocyst. (**a**) In the Sporozoite stage, *Plasmodium* infection affects the fitness and blood feeding of mosquitoes. Most of the experiments reported here are laboratory-based. Host-seeking and biting are the only behaviours on which field experiments have been performed. (**b**) The oocyst stage of *Plasmodium* impacts the fitness and blood feeding of mosquitoes. Most of the oocyst stage-based experiments reported here are also laboratory-based. Field data are only available for changes in fecundity.

The malaria parasite *Plasmodium* affects the fitness of female *Anopheles* mosquito differently during sporozoite (Figure 1a) and oocyst (Figure 1b) development stages. Fitness involves behaviours such as flight, mating, fecundity, and reproduction capacity, and a wide range of studies have been conducted on *Plasmodium*’s ability to alter its vector’s fitness. Researchers [19] focused on the changes in flight activity of *An. stephensi*-infected *P. cynomolgi*, which can cause disease in monkeys, and found a reduction in the distance flown, flight speed, length of initial flight, and longest flight. Interestingly, the impact on mosquitoes’ flight activity was also found to be maximum when rodent malaria parasite *P. yoelii* completed the oocyst development phase and sporozoite reached the salivary glands [20].

Fecundity (number of eggs laid) is another attribute that changes with pathogen infection. Low egg production was observed in *An. stephensi* after infection with *P. yoelii nigeriensis*. Moreover, the reduction was more noticeable when the bloodmeal contained malaria gametocytes and *An. stephensi* developed oocysts [21]. Although the decrease in the egg number may have been due to reduced blood feeding, their later study showed that the reduction in fecundity could not have been only due to reduced bloodmeal size [22]. Hacker [23] analysed the influence of *P. gallinaceum* on six different location-based strains of *Ae. aegypti* and demonstrated that the parasite reduced the egg production in all six strains. However, the amount of impact was different in all strains and was based on the degree of parasite infection density. The researchers also analysed the impact on the fecundity of *Ae. aegypti* at lower and higher densities of *P. gallinaceum* in mosquitoes. A reduction in fecundity was noticed at lower densities while it did not change further at higher densities [24]. It should be noted that *Ae*. *aegypti* is not considered a vector for *P*. *gallinaceum*. The change in fecundity primarily depends on the midgut proteases and gonotrophic cycles [25]. Hogg and Hurd [22] observed the reduction in fecundity in *P. yoelii nigeriensis*-infected *An. stephensi* during three successive gonotrophic cycles and recorded the highest impact during the first cycle and lowest during the third cycle. In their work, they concluded that the reduction in fecundity depends on the density of the parasite; the infection intensity was high during the first cycle and reduced by the third cycle. This research was conducted in controlled laboratory conditions. In 1997, researchers caught wild mosquitoes infected with *An. gambiae* and examined their fecundity; the results were similar to the laboratory experiments, with a 17.5% reduction in egg production [26]. The impact of the parasite on the next generation’s fecundity was ignored until Ahmed et al. performed laboratory experiments using *P. yoelii nigeriensis*-infected *An. gambiae*. The results showed that the fecundity was also significantly reduced in the next generation [27]. Researchers [28] analysed the impact of *P. chabaudi* on the fecundity of *An. stephensi* and showed that parasite genetics correlated with reduced fecundity, mainly due to reduced bloodmeal size. The researchers also showed that mosquitoes that fed on mice, which were infected with more virulent strains of the parasite, were more fecund. These results may suggest the shaping of malaria parasite population genetics towards more virulence.

Female mosquitoes need to have high fertility to hatch the maximum number of larvae from their eggs. The interaction of parasite–vector also affects the fertility (number of eggs hatched) of the mosquitoes. Researchers (Freier and Friedman, 1976) spotted a lower reproductive capacity in *P. gallinaceum*-infected *Ae. aegypti* compared to the uninfected. Moreover, fertility reduced significantly in successive infected generations [27], with a 61.8% reduction in the fertility of *P. yoelii nigeriensis*-infected *An. gambiae* in comparison with the previous generation.

Overall, the research suggests that infection with malaria parasites reduces fecundity and fertility of mosquitoes; however, the degree of reduction depends on the genetic strain of the parasite, which may drive evolution towards more virulence. There also appears to be changes in fecundity depending on the developmental stage of the parasite.

#### 2.1.2. Changes in Blood Feeding

Mosquito blood feeding attributes involve attraction (host-seeking) and biting (rate, duration). Rossignol et al. used a *P. gallinaceum*-infected *Ae. aegypti* and observed the behavioural alterations for five days during the sporozoite stage. Their research found that olfactometer response when exposed to an anaesthetised guinea pig, as a measure of host-seeking, increased in infected mosquitoes [29]. The developmental stage of the parasite plays a significant role in defining its impact on the vector. Anderson et al. performed a laboratory-based experiment and examined the impact of *P. yoelii nigeriensis* on *An. stephensi* during different developmental stages of the parasite and concluded that feeding preference towards the human host decreased during the oocyst stage and increased when there were transmissible sporozoites in the salivary glands [30]. Cator et al. also confirmed these results, showing that mosquito’s biting preference increased during the infectious stage of the malaria parasite, possibly for increasing the transmissibility of the parasite [31].

To understand whether the attraction of mosquitoes towards human skin odour changes after infection, researchers [32] performed a laboratory-based experiment and noticed a higher attraction towards human skin odour in *An. gambiae sensu stricto* after the ingestion of *P. falciparum*-infected human blood. Similar results were found in lab-based research performed by Stanczyk et al. in 2019. They observed that *P. falciparum*-infected *An. gambiae*, *P. berghei*-infected *An. stephensi*, and *P. berghei*-infected *An. gambiae* have different responses towards human odour [33]. The difference in attraction could be because different types of mosquitoes and different parasite-infected mosquitoes have a different threshold of attraction [34]. Another question that can arise is whether the desire to seek out a second blood meal is altered in malaria-infected mosquitoes. Ferguson and Read answered this question in their research, wherein they showed that compared to uninfected *An. stephensi*, almost one and half times more blood-seeking behaviour was shown in infected *An. stephensi* towards *P. chabaudi*-infected mice [35].

Several experiments have been performed to examine the probing time, frequency, and ability to locate blood. For example, researchers [36] inspected the field-collected *An. gambiae sensu lato* and *An. funestus* mosquitoes infected with *P. falciparum* and found a higher probing rate in both compared to the uninfected mosquitoes. Similar results were observed by other researchers using different systems [37,38]. The malaria parasite also damages the mosquito’s salivary glands, which may ultimately increase the probing time and affect the ability to locate the blood vessels [39]. This may potentially increase infective host contacts, increasing transmission potential.

Apart from feeding persistence, biting rates can also differ during different developmental stages of the parasite. Previously, researchers [40] witnessed a low biting rate in *Ae. aegypti* during the oocyst stage of *P. gallinaceum* and a higher biting rate during the transmissive sporozoite stage. The lower feeding persistence towards the human host and biting rates during the oocyst stage may be because the parasite cannot be transmitted during the development period (oocyst stage). For transmission to occur, the ideal situation is to allow mosquitoes to survive during the parasite development period to transmit successfully in later stages [41]. The number of bites is a crucial factor impacting mosquitoes’ life span (Anderson and Brust, 1996); therefore, the parasite may modulate mosquito’s behaviour to survive during the oocyst stage by reducing its number of bites. However, it increases the number of bites or biting duration during the sporozoite stage to maximise its spread.

Researchers [42] analysed the impact of human-sourced *P. falciparum* (sporozoite stage) on *An. gambiae* during the night and observed a decrease in blood obtaining efficiency and increased feeding activity. The increase in feeding activity can consist of fewer feeds of longer duration or multiple feeds of short duration [40]. In the case of fewer feeds of longer duration, it can increase the chance of successful infection. On the other hand, in the second case, the mosquito probes multiple times, increasing the chance of the parasite spread by infecting more hosts. Several mosquito species, including *An. punctulatus*, feed more than once during a single gonotrophic cycle [43]. A group of researchers performed a similar kind of field experiment by using *An. punctulatus* mosquitoes and *P. falciparum* and *P. vivax* parasites during the different times at night and observed higher values of mean blood volume in infected mosquitoes with no dependence on time, while the uninfected mosquitoes had a steady increase in mean blood volume during the night [44]. The higher blood volume in infected mosquitoes may instigate the rapid spread of the parasite, while the steady increase in the mean blood volume of uninfected mosquitoes could be due to people sleeping at the time, with fewer chances of mosquitoes getting disturbed while feeding.

#### 2.1.3. Adaptation to Insecticide Treated Nets

With increased insecticide resistance [45] and disappointment of the global malaria eradication program by the WHO [46], insecticide-treated nets (ITNs) garnered the attention of health experts when studies examined the effect of pyrethroid insecticides on the traditional mosquito nets for reducing the exposure to malarial vector [47,48]. Given their safety and effectiveness, ITNs have become the most powerful malarial control tool, becoming an important part of many global malarial control strategies. After the ITNs became popular, scientists also started measuring the impact of ITNs on the behaviour of infected mosquitoes. Bockarie and Dagoro (2006) performed field-based experiments using different *Plasmodium* and mosquito species and compared the attraction of *P. vivax*- and *P. falciparum*-infected *An. punctulatus* towards people on Lihir Island (Papua New Guinea) before and after introducing the ITNs. In the case when ITNs were not introduced, the proportion of *P. falciparum*-infected mosquitoes biting people was higher than *P. vivax*. However, after the adoption of ITNs, the proportion of *P. vivax* biting people (before they return to bed under the protection of bed nets) was increased significantly compared to *P. falciparum* [49], exposing more people to *P. vivax* than *P. falciparum*. The change before and after introducing the ITNs could have been due to the different biting behaviours caused by different parasite species. This may partly explain the shift from *P. falciparum* to *P. vivax* infections after the introduction of ITNs. *P. vivax*-infected mosquitoes have a tendency to bite earlier compared to *P. falciparum*-infected mosquitoes. The duration of sporogony for *P. vivax* is five days, while for *P. falciparum* it is nine days [50]. Therefore, *P. vivax*-infected females are on average younger than *P. falciparum* females, and young females have the tendency to bite earlier than old females [51].

### 2.2. Arbovirus and Behavioural Modulations in Mosquitoes

Arboviruses such as dengue, Zika, chikungunya, West Nile, and La Crosse are spread through a cycle of transmission involving mosquitoes and humans or animals. Dengue viruses (DENVs) are transmitted by *Aedes* mosquitoes [52] and consist of four serotypes, namely, DENV-1, DENV-2, DENV-3, and DENV-4 [53]. *Ae. aegypti* is a known vector for ZIKV transmission. *Aedes* mosquitoes also transmit Chikungunya virus (CHIKV), an alphavirus (Robinson, 1955).

#### 2.2.1. Changes in Fitness

**Figure 2:**
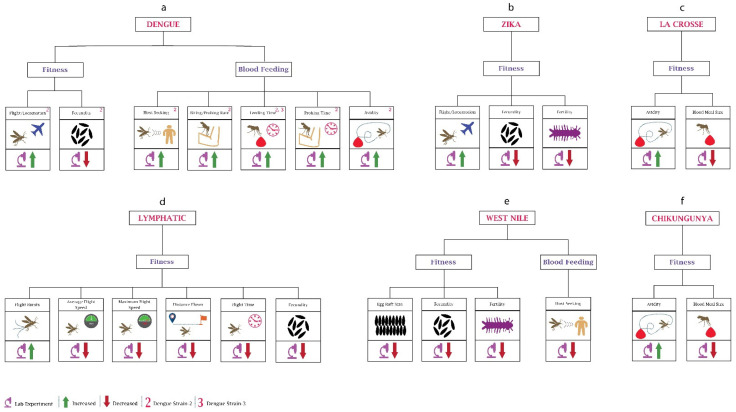
Behavioural changes in mosquitoes due to viruses: (**a**) In dengue, the majority of the work has been performed on the blood-feeding behaviour. Most of the data are based on research using DENV-2. DENV-3 has been used to determine changes in feeding time. (**b**) In Zika, studies have been only performed on the mosquito’s fitness. (**c**) Mosquito fitness changes after infection with La Crosse virus. (**d**) Mosquito behavioural changes after infection with lymphatic filariasis is shown here. (**e**) In the case of the West Nile virus, little work has been performed on both fitness and blood feeding. (**f**) Chikungunya virus infection leads to changes in the avidity and blood feeding size of the mosquitoes.

Although vertical dengue transmission has been reported [54], for mosquitoes, the primary source of DENV is during human blood feeding. Several studies have observed the impact of different dengue serotypes on the behaviour of mosquitoes (Figure 2a). When researchers [55] infected *Ae. aegypti* female mosquitoes with dengue serotype (DENV)-2, they observed an increase of around 50% in infected mosquitoes’ locomotor activity in contrast to uninfected mosquitoes. Other researchers [56] recorded higher activity in DENV2-infected *Ae. aegypti* during the night-time compared to the daytime. Although not supported by any studies, this higher locomotor activity could potentially be responsible for increasing the chance of finding a suitable host. Another group of researchers noticed a change in behaviour depending on the progression of infection within the mosquito, with increased locomotion during 4–6 days post-infection, increasing spatial exploration and avoiding predation. However, 14–16 days after DENV infection, *Ae. aegypti* mosquito activity was reduced and host-seeking ability was increased, increasing the risk of virus transmission [57]. From the increased locomotor activity, it can also be inferred that DENV may also enhance the biting rate of mosquitoes, as supported by another study [58], where researchers using a mathematical model observed a higher biting rate in dengue-infected *Ae. aegypti*. Research (Gaburro et al., 2018b) has also found that DENV-2 changes the olfactory oviposition preferences of skatole reared *Ae. Aegypti*, indicating that infected mosquitoes may choose oviposition sites further away, possibly increasing transmission risk. Apart from the olfactory oviposition preference, DENV infection also has been shown to reduce fecundity (Maciel-de-Freitas et al., 2011).

Like DENV, ZIKV also modulates the behaviour of its host vector for transmission (Figure 2b). A group of researchers [56] noticed a significantly increased diurnal locomotion activity in ZIKV-infected *Ae. aegypti* in contrast to uninfected mosquitoes, especially during the egg-laying period, suggesting that this may increase the spread of ZIKV either by mosquito bites or due to vertical transmission of the virus. In contrast, Padhila and co-authors [59] found a lower locomotion activity in infected *Ae. Aegypti* which can be explained by the fact that the mosquitoes were kept isolated from host odours and inter-mosquito communication. The infection source was also different, which may have had some impact on the fecundity. *Ae. aegypti* females lay approximately 20 to 140 eggs per blood meal depending on females’ fecundity, blood meal size, body size, and reserves [12,60]. Researchers [61] analysed the egg production per clutch and egg production per microlitre of blood ingestion and found that in ZIKV-infected *Aedes*, the number of eggs laid per female during the later clutches was increased while the egg production per microlitre of blood ingestion remained stable. This shows that blood ingestion in ZIKV-infected *Ae. aegypti* increases with age, increasing the number of eggs per clutch. Resck et al. investigated the effects of ZIKV infection on the oviposition efficiency of *Ae. aegypti* females and compared them with CHIKV-infected females. They observed that the infertility decreased in CHIKV-infected females from 3.8% to 2.7% in first and second gonotrophic cycles, respectively, while it was increased in ZIKV-infected females from 2.1% to 6.8% during the first and second gonotrophic cycles, respectively, and it increased further with age [11].

Since its first isolation in 1937 [62], some work has been done to determine the impact of WNV on vectors’ behaviour (Figure 2e). Researchers [63] have noticed several changes in the WNV infected *Culex tarsalis*, including lower fecundity during first oviposition (50% reduction), smaller egg rafts (150 eggs per raft for uninfected vs. 110 eggs per raft for infected), and lower egg hatch rates. The research suggests that WNV infection reduces the fitness of mosquitoes affecting their progeny.

Researchers investigated the effects of CHIKV (Robinson, 1955) infection on the fertility of young and old *Ae. aegypti* females and concluded that fertility was significantly decreased in both young and old CHIKV-infected females, reducing the viability of their eggs [11]. Another group [64] focused on the time before egg laying of CHIKV-infected *Ae. albopictus* and found that CHIKV significantly shortened the time before oviposition.

#### 2.2.2. Changes in Blood Feeding

By inspecting biting behaviour and monitoring the feeding of *Ae. aegypti* during the second and third weeks post-infection, researchers observed that blood feeding time had a direct relationship with the days post-infection, with infected mosquitoes taking more time to blood feed from mice during the second and third weeks post-infection [65]. Researchers also examined the biting duration and probing period of *Ae. aegypti* mosquitoes infected with DENV-3 [66] and found that infected mosquitoes take a longer time to obtain a blood meal than uninfected mosquitoes, increasing the probability of virus transmission. The reason behind increased probing and feeding times was found to be the modulation of key genes in the olfactory organ, the antenna [67]. Another group of researchers [10] observed that DENV-2 reduces the motivation to feed (possibility of obtaining blood meal and blood meal size) but increases the avidity (possibility of refeeding after the interruption in the first meal) of *Ae. aegypti*.

Researchers have focused on the host (birds)-seeking behaviour of infected *Culex pipiens* and found that WNV reduced the host-seeking behaviour by about threefold due to its effect on the central nervous system of mosquitoes [68]. This shows that WNV infection does not favour transmission by reducing mosquitos’ host-seeking behaviour.

La Crosse encephalitis virus (LACV) belongs to the family *Bunyaviridae* and is one of the most underreported vector-borne diseases causing encephalitis among children in the United States. LACV significantly impacts the blood feeding behaviour of mosquitoes (Figure 2c). The researchers found that the infection with LACV decreases the blood meal size of both *Ae. triseriatus* and *Ae. albopictus*, while the avidity (refeeding rate) was increased for *Ae. triseriatus* and remained constant for *Ae. albopictus* [69]. Another group of researchers obtained similar results in their experiment; 21% of infected *Ae. albopictus* took a partial blood meal in a single probe compared to uninfected mosquitoes where 52% of females were fully engorged after the first probe. Moreover, 79% of infected females were probed multiple times for partial engorgement compared to uninfected ones, where 48% females probed multiple times for full engorgement [70]. LACV’s ability to increase the refeeding rate may also increase the transmission rate.

### 2.3. Lymphatic Filariasis (LF) and Behavioural Modifications in Mosquitoes

Lymphatic filariasis is a parasitic disease caused by microscopic nematode and is transmitted by wide range of mosquito species.

#### 2.3.1. Changes in Fitness

Evidence from experimental infection studies showed that LF could impact the fitness of *Ae. aegypti* mosquitoes (Figure 2d). Using tethered flight mill, researchers [71] found that *Brugia malai* infection significantly reduced the flight distance, average flight speed, and maximum flight speed but increased the number of flight bursts. The results showing a detrimental effect on mosquito flight may explain the heterogeneous distribution of lymphatic filariasis, which poses a challenge for elimination. A flight mill-based study conducted in 1975 also found that the domestic animal parasite *Burgia pahangi* notably reduces the flight length and overall flight time of *Ae. aegypti*, and the number of infected mosquitoes that were unable to fly was also higher compared to uninfected mosquitoes [72]. Interestingly the number of non-flying mosquitoes increases with the development of the *Burgia pahangi* parasite [73]. Lymphatic filariasis also reduces the fecundity of *Ae. aegypti* [74]. Gleave et al. also found that reduction in fecundity was density-dependent; parasite density had an inverse relation with fecundity. The reduction in fecundity can be due to nutrient competition because as the parasite develops, it extracts more energy from the host mosquito. Thus overall, LF infection leads to a detrimental effect on the fitness of mosquitoes, leading to its heterogeneous spread.

#### 2.3.2. Changes in Blood Feeding

Although blood feeding is a critical behavioural aspect, limited research has been conducted on the feeding behaviour of LF infected mosquitoes. Similar to *Plasmodium*, LF reduces the mosquitoes’ host-seeking behaviour during the development phase, which increases significantly with parasitic development [74]. An increase in host-seeking behaviour during the early development phase does not benefit the parasite transmission and can shorten the life span of the vector host. An increase in host-seeking behaviour late in the development phase can increase its transmission.

## 3. Behavioural Changes in Ticks after Infection

Ticks are parasitic arachnids, and they belong to phylum Arthropoda and subphylum Chelicerata. Most of ticks have four life stages: egg, six-leg larva, nymph, and adult [75]. Usually after hatching from the eggs, ticks feed on blood at every stage to molt and develop. Depending on species, ticks usually take about three years to complete their life cycle, and blood feeding is a critical factor during this time. Ticks that do not find a host for the next feed can die before completing their life cycle [76]. Ticks at different life stages can feed on different hosts, including mammals, birds, and amphibians, transmitting pathogens between these hosts during feeding (Table S1).

### 3.1. Borrelia Bacteria and Behavioural Modulations in Ticks

*Borrelia* is a bacterium that belongs to the phylum Spirochaetes and the family *Spirochaetaceae* [77]. It is transmitted through *Ixodes* ticks and can cause Lyme disease. Ticks become infected with *Borrelia* spp. if they feed on the infected host, usually mammals. *Borrelia* can only be transmitted to the host during the nymph and adult stages of ticks [78].

#### 3.1.1. Changes in Fitness

**Figure 3:**
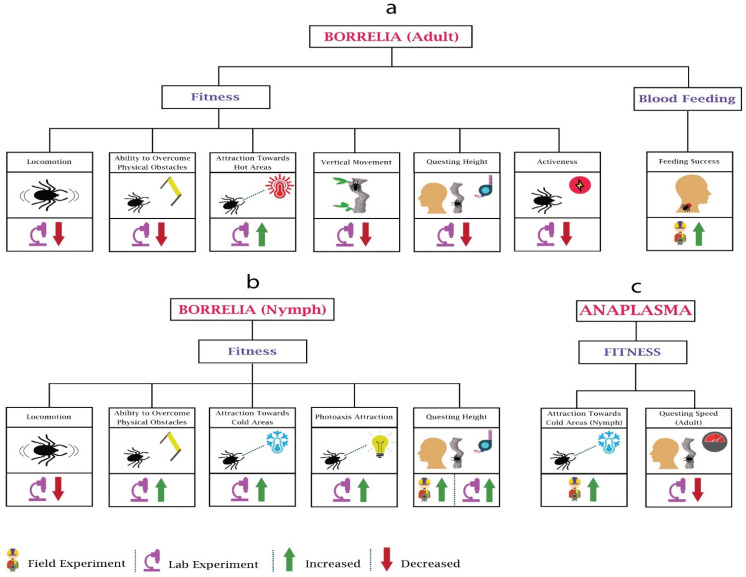
Behavioural changes in ticks (adult, nymph) due to *Borrelia* and *Anaplasma*: Tick-borne diseases’ pathogens impact the fitness and blood feeding of the ticks. (**a**) In *Borrelia*, the majority of work has been performed on the fitness. (**b**) Contrary to adults, in *Borrelia*-infected nymphs, ability to overcome physical obstacles, attraction towards cold areas, and questing heights increases. (**c**) In *Anaplasama*, research has only been performed on nymphs’ attraction towards cold areas and questing speed of adults.

Whenever *Borrelia* infects a tick, it brings about some distinct alterations in the behaviour of ticks, some of which support the tick survival and enhance the transmission of *Borellia*. Several studies have been performed to determine the impact of *Borrelia* on the behaviour of adults (Figure 3a) and nymphs (Figure 3b). Researchers [79] investigated the locomotor activity of adult and immature *Ixodes ricinus* infected with *Borrelia burgdorferi* and found a decline in the locomotion of both after the infection. Other researchers [80] found higher questing heights among the *Borrelia burgdorferi*-infected *Ixodes persulcatus* compared to the uninfected. The research performed by Lefcort and Durden on *Ixodes scapularis* ticks determined that compared to the uninfected adults, *Borrelia burgdorferi*-infected adults were less fit to tackle physical obstacles and vertical surfaces and quested at lower heights. This may reduce their ability to find potential mates as well as reduce their exposure to predators. In contrast, their research observed the changes in nymphs, such as increased questing heights, greater tendency to overcome physical obstacles, higher phototaxis attraction, and increased attraction to vertical surfaces [81]. This may increase the probability of contact between the tick and potential hosts. These apparent differences in behaviour changes may be explained by the needs of the life stages. Nymphs are required to blood feed within weeks to few months, while adults can survive without blood feeding for much longer. This means that it is of an evolutionary advantage to the bacteria if nymphs have increased questing heights leading to more chance of contact with hosts. On the other hand, allowing the adult to engage in a more conservative host-seeking behaviour, increasing their survival, may also be advantageous to the bacteria for transmission when conditions are favourable.

Another group of researchers examined *Borrelia burgdorferi*-infected nymphs of another species, *Ixodes pacificus*, and observed higher densities on logs and trunks than in the leaf litter [82]. This may be of significance since the nymphs of the principal hosts for these nymphs—lizards, white-footed mice, and eastern grey squirrels—have direct contact with logs. Alekseev and Dubinina observed significantly higher proportions of *Borrelia*-infected nymphs at lower temperatures between temperatures of 10 and 14 °C, and while the number was reduced significantly at slightly high temperatures (15–20 °C), no infected nymph was found at higher temperature (21–26 °C). Interestingly, they also observed that after infection, adults become more tolerant of higher temperatures as compared to the infected nymphs [83]. Herrman and Gern observed the horizontal walk distances of *Borrelia spirochetes*-infected *I. ricinus* nymphs in humidity gradients and concluded that infected nymphs were less likely to walk on the horizontal plane, and when they did move, they moved for shorter distances. In their research, they also observed that uninfected low-fat nymphs preferred to walk in higher humidity (area with water vapour saturation), while the high-fat uninfected nymphs tended to move towards low humidity [84]. Later on, the same group of researchers confirmed the presence of higher fat among the *Borrelia* spirochetes-infected *Ixodes ricinus* compared to the uninfected [85]. On this basis, it can be hypothesised that *Borrelia* spirochetes infection may increase the fat energy source in *Ixodes ricinus* nymphs, which increases their tolerance to desiccation, and as a result, they had higher survival under desiccating conditions.

#### 3.1.2. Changes in Blood Feeding

A study showed greater attraction of *Ixodes ricinus* nymphs towards *Borrelia afzelii*-infected bank voles (*Myodes glareolus*) and higher body weight of infected nymphs after feeding in contrast to the uninfected nymphs [86], possibly related to increased bloodmeal size or increased fat reserves. Faulde and Robbins performed a study using human volunteers and white cotton dragging blankets to see the host-finding efficacy of infected female *Ixodes ricinus*, finding a higher infestation rate of *Borrelia burgdorferi*-infected adult females on human volunteers compared to the cotton blanket [87]. This indicates increased transmission potential of *Borrelia* by infected ticks, either by increased host-seeking or bloodmeal size.

### 3.2. Anaplasma and Behavioural Modification in Ticks

*Anaplasma*, which causes granulocytic anaplasmosis, is another tick-transmitted bacterium. It belongs to the order Rickettsiales and the family *Anaplasmataceae* [88]. Although it is mainly transmitted by the bite of infected ticks, it can also be transmitted during organ transplants and transfusion [89].

#### 3.2.1. Changes in Fitness

*Anaplasma* is the most widespread tick-borne infectious agent in animals in Europe [90]. Despite the importance, limited work has been done on the impact of *Anaplasma* on the behaviour of ticks (Figure 3c). Researchers [91] observed that *Anaplasma phagocytophilum* induces the expression of IAFGP (*Ixodes* antifreeze glycoprotein) in *Ixodes scapularis* nymphs, which helps them survive in the cold regions of the United States; hence, more infected ticks can be found in cold areas compared to uninfected. The researchers in [92] analysed the questing speed of *Anaplasma phagocytophilum*-infected *I. scapularis* at 4 °C, 22 °C, and 37 °C compared to uninfected ticks and found that the questing speed of infected ticks was reduced approximately 30–60% due to the upregulation of hsp20 and downregulation of hsp70 proteins. Infected ticks spreading to new areas helps pathogens to spread widely, e.g., *Anaplasma phagocytophilum* increases the colonisation of infected *Ixodes scapularis* to newer geographical areas.

#### 3.2.2. Changes in Blood Feeding

*Anaplasma* modulates the immune response of ticks, but it does not affect the feeding behaviour, which helps to maintain the vector capacity [93]. However, there is a need for more work to be done on feeding and other ticks’ behaviours.

### 3.3. Babesia and Behavioural Modification in Ticks

*Babesia* is a parasite that affects the host’s blood cells and is transmitted through ticks [94]. During the blood ingestion, sporozoites enter the mammalian host such as mice. These enter erythrocytes and undertake asexual reproduction. Some of these pathogens differentiate into male and female gametes. Once these pathogens are ingested by ticks during blood feeding, the fusion of gametes helps them to undergo a sporogonic cycle and yield sporozoites [95], which are then transmitted to the susceptible host via subsequent blood feeding by the tick.

#### 3.3.1. Changes in Fitness

**Figure 4:**
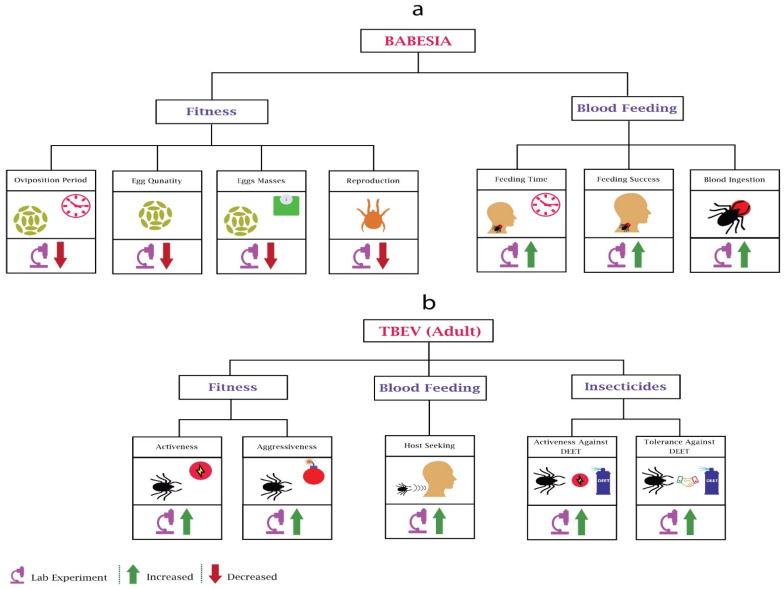
Behavioural changes in ticks due to Babesia and TBEV: (**a**) In Babesia’s infected mosquitoes’ fitness, most of the work performed was related to oviposition and reproduction. Babesia’s infection reduces the oviposition period, egg quantity, egg masses, and reproduction of the ticks. In blood feeding, Babesia increases the feeding time, feeding success, and blood ingestion. (**b**) In all TBEV-based experiments, only adult ticks were considered. In fitness, TBEV infection increases the activeness and aggressiveness of ticks. In blood feeding, TBEV increases the host-seeking of the ticks. In adaptations against insecticides, TBEV increases the activeness and tolerance against DEET.

*Babesia* significantly impacts the fitness and blood feeding of adults and nymphs (Figure 4a). Studies have provided insight into the fecundity of *Babesia Bovis*-infected *Boophilus microplus* females, where infection significantly reduced the egg quantity, egg mass, and oviposition period [96]. Another parasite species, *Babesia bigemina,* also reduced the egg production of *Boophilus microplus* [97]. These reductions could be due to the nutrition competition between parasites and ticks. Researchers [98] also observed that that reproduction of piroplasm *Babesia bigemina*-infected *Boophilus decoloratus* was reduced significantly due to a decrease in egg production.

#### 3.3.2. Changes in Blood Feeding

Researchers observed the feeding success rate of *Babesia microti* infected *Ixodes trianguliceps* nymphs [99] and concluded that infection increases the feeding success; however, feeding success was not associated with the pathogen density in vectors. Another group of researchers used the same parasite species for a different tick species, *Ixodes scapularis*, and observed a host-dependant increase in feeding time and engorgement [100].

### 3.4. Tick-Borne Encephalitis Virus (TBEV) and Behavioural Modulations in Ticks

Tick-borne encephalitis virus (TBEV) belongs to the family *Flaviviridae* and is a single-stranded RNA virus [101]. Three subtypes of the virus are European TBEV, Siberian TBEV, and Far Eastern TBEV [102]. The virus infects the ticks and can be transmitted trans-stadially and trans-ovarially [103]. It can also be transmitted by drinking raw milk of infected animals [104].

#### Changes in Fitness

Despite its importance, our knowledge about the impact of tick-borne encephalitis virus (TBEV) on ticks is very limited (Figure 4b). Researchers evaluated the behaviour of TBEV-infected *Ixodes ricinus* ticks [105] and found that infected ticks were more aggressive and active, being found very often on humans. The results also showed that infected ticks were tolerant and active against repellent *N*,*N*-diethyl-meta-toluamide (DEET). The only research found on questing height did not find any behavioural change compared to uninfected ticks [80]. However, they used a different species, *Ixodes persulcatus sch*. The increasing prevalence of TBEV across human-preferring ticks [106] requires more research on behavioural modifications of TBEV.

## 4. Conclusions

In this review, we discussed various behavioural modulations in vectors due to infection by bacteria, viruses, or parasites. Although the mechanism of this behavioural change is not well understood, this review helps in understanding virus–vector interactions aiding improved epidemiological models, developing efficient mosquito traps, and devising new vector management strategies.

There is a long history of epidemiological models, starting from Ross’s ordinary differential equation-based model [107]. Most of the models since developed have followed Ross’s theme and considered population as a determinist factor [108,109,110]. Although the new emerging stochastic models included individuals’ impact, they rarely considered more nuanced behaviours such as feeding frequency, fecundity, and the host-seeking behaviour [111,112]. A review of 325 different epidemiological model-based publications observed that the models that considered mosquito ecology, seasonality, mosquito and host behaviour, and pathogen evolution failed to consider the heterogeneous vector biting and encounter between vector and host [113]. Heterogeneous exposure can generate disease hotspots and is a critical factor in designing disease control intervention [114]. In this review, vectors’ behaviours are categorised on the basis of species, which may help in modifying existing models with the inclusion of features based on their species and multiple behaviours. Moreover, researchers would be able to perform better epidemiological simulations based on variations in fecundity, biting frequency, reproduction, and host-seeking.

When looking for a host for feeding, vectors follow different cues such as released carbon dioxide (CO_2_), skin odour, and body heat [115]. Considering this behaviour, several mosquito traps have been developed that use a blend of carbon dioxide, host-mimicking odour, visual signs, and airflow to entice and catch mosquitoes [116,117]. However, currently available mosquito traps have varying performance in different areas against different species [118]. Therefore, trap designs based on specific flight behaviour, probing behaviour, and avidity will help in attracting specific mosquitoes, possibly only infected mosquitoes. This knowledge will also help determine a location for trap deployment for improved surveillance and detecting infected mosquitoes.

To reduce the burden of vector-borne diseases, researchers have adopted different vector management strategies. These strategies include removing vector habitats, structural barriers, control of adult vectors, and control in the larval phase [119]. Control during the adult and larval phase demands biological, behavioural, physical, and chemical control measures [120]. Insecticide-treated bed nets are employed to control the mosquito bites physically, while pesticides are used to control the mosquito population chemically. According to recent research, vectors are developing resistance to current insecticides and are adopting new behaviours to help them avoid ITNs [121]. Moreover, the factors that increase the vector’s fitness in a specific region or environment are poorly understood. Knowledge in vectors’ behavioural change will help industries in formulating new pesticides on the basis of their changing behaviours. It will also help public health authorities in the application of these insecticides according to vector behaviour to target a large population. It will also help in recognising the behaviours during larva, pupa, and adult stages and identifying the factors that impact the vector’s fitness.

Given the spectrum of the behavioural changes, it remains to be seen whether these changes are deliberate modifications effected by the pathogen in order to increase its transmission or fitness. As reviewed here, malaria parasite infection causes a reduction in the flight behaviour, fecundity, and fertility of mosquitoes. It may appear that the parasite infection is leading to reduced fitness in a mosquito, which is counter-intuitive. However, the effect was found to be dependent on malaria parasite genotype, with more virulent strain causing increased fecundity due to increased bloodmeal, possibly driving malaria evolution towards more virulence. It was also seen that infection with malaria infection leads to decreased host-seeking during a non-transmissible stage of the parasite, while increased host-seeking and mosquito biting was found during an infectious stage of the parasite, increasing transmission probability. Similar results have been shown in the case of dengue-infected *Aedes* mosquitoes, with increasing host-seeking during an infectious stage of the virus cycle. Lymphatic filariasis infection, on the contrary, showed a detrimental effect on flight behaviour, which may explain the heterogeneous distribution of the disease. We also discuss the impact of tick-borne pathogens on their vector, and interestingly, the behavioural effect is dependent on the developmental stage of ticks, with infected nymphs engaging in more risky behaviour, while infected adult ticks engage in more conservative host-seeking behaviour, in order to increase survival. The research suggests that the nuances of behavioural changes should be considered in the context of host ecology, pathogen transmission, disease epidemiology, and environmental conditions to make sense of their impact. Some of the implications of the observed behavioural change on disease epidemiology remain hypothetical.

Despite this research on the behavioural impact of infections in vectors, molecular mechanisms underlying these behavioural changes remain largely unknown. Previous research has determined genes associated with various insect behaviours such as olfaction [122], host-seeking [123], blood-feeding [124], and egg-laying [125]. It remains to be seen whether pathogens infecting these vectors alter expression of these genes, leading to behavioural changes. Infectious diseases such as Zika mainly affect the host brain; research has found that mammalian neurons die off due to ZIKV-caused by glutamate-induced excitotoxicity [126], while mosquito neurons show increased glutamate release but survive the toxicity [56]. As mentioned previously, mosquitoes infected with ZIKV also modify their behaviour, such as through increased activity, which may be attributed to release of the excitatory neurotransmitter glutamate. Studies using appropriate models and novel technologies, which can be standardised, are needed to further our understanding.

Overall, this review discusses changes in the behaviour of vectors in response to infection. There is a need for further research in this area, which will be not only important in vector-borne disease control but also the basic understanding of vector biology and vector–virus interaction.

## Supplementary Materials

The following are available online at https://www.mdpi.com/article/10.3390/pathogens10111376/s1, Table S1: Vector-borne diseases and vector behavioural changes.
