# Supplementary material for: Advances in Understanding Vector Behavioural Traits after Infection
Source: Pathogens. 2021 Oct 24;10(11):1376. doi: 10.3390/pathogens10111376 (PMC8621129; doi:10.3390/pathogens10111376)
Supplement: Supplementary file 1 [file pathogens-10-01376-s001.zip › pathogens-1398013-supplementary.pdf]

Table S1: Vector-Borne Diseases and Vector's Behavioural Changes

Vector	Virus/Parasite	Vector Specie	Bacteria Type	Infection Source	Field/Laboratory	Behavioural Change	Reference	Virus/Parasite Stage	Remarks
Mosquitoes									
	Malaria								
		Anopheles Punctulatus	*Plasmodium Falciparum* & *Plasmodium Vivax*	Infected Humans	Field	Enhance blood-feeding	(Koella and Packer, 1996)	Sporozoite	
		Anopheles Gambiae	*Plasmodium Falciparum*	Infected Humans	Field	Decreased efficiency in obtaining blood and increased feeding activity	(Anderson et al., 2000)	Sporozoite	
		Anopheles Gambiae	*Plasmodium Falciparum*	Infected Humans	Field	Increased the frequency of multiple feeding	(Koella et al., 1998)	Sporozoite	

		Anopheles Stephensi	*Plasmodium Yoelii Nigeriensis*	Infected Mice	Laboratory	Feeding persistence towards human host decreased	(Anderson et al., 1999)	Oocyst	
		Anopheles Stephensi	*Plasmodium Yoelii Nigeriensis*	Infected Mice	Laboratory	Feeding persistence towards human host increased	(Anderson et al., 1999)	Sporozoite	
		Aedes Aegypti	*Plasmodium Gallinaceum*	Infected Chicken	Laboratory	Decreased bitting rate and blood feed volume	(Koella et al., 2002)	Oocyst	
		Aedes Aegypti	*Plasmodium Gallinaceum*	Infected Chicken	Laboratory	Increased bitting rate and blood feeding volume	(Koella et al., 2002)	Sporozoite	

		Anopheles Stephensi	*Plasmodium Chabaudi*	Infected Mice	Laboratory	Super attraction towards the host	(Ferguson and Read, 2004)	Sporozoite	
		Anopheles Punctulatus	*Plasmodium Vivax*	Human	Field	Increased host seeking	(Bockarie and Dagoro, 2006)	Sporozoite	
		Anopheles Gambiae Sensu Stricto	*Plasmodium Falciparum*	Human Blood	Laboratory	Attraction towards human skin odour increased	(Smallegange et al., 2013)	Sporozoite	
		Anopheles Stephensi	*Plasmodium Yoelii*	Infected Mouse	Laboratory	Reduced attraction towards host	(Cator et al., 2013)	Oocyst	
		Anopheles Stephensi	*Plasmodium Yoelii*	Infected Mouse	Laboratory	Increased attraction towards host	(Cator et al., 2013)	Sporozoite	
		Aedes Aegypti	*Plasmodium Gallinaceum*	Infected Guinea pig	Laboratory	Decrement in Egg output and	(Rossignol et al., 1986)	Sporozoite	

						Increment in probing rate (If host-vector contact duration is less)			
		Anopheles gambiae,<br><br>*Anopheles Stephensi*	*Plasmodium Falciparum,*<br><br>*Plasmodium Berghei*	Infected Mice	Laboratory	Change in Olfactory responses	(Stanczyk et al., 2019)	Sporozoite	
		Anopheles Gambiae Sensu Lato<br><br>&<br><br>*Anopheles Funestus*	*Plasmodium Falciparum*	Human	Field	Increased probing rate	(Wekesa et al., 1992)	Sporozoite	

		Aedes Aegypti	*Plasmodium Gallinaceum*	Infected Guinea pig	Laboratory	Impaired the vector's ability to locate blood vessels	(Rossignol et al., 1984)	Sporozoite	
		Anopheles Stephensi	*Plasmodium Cynomolgi*	Rhesus monkeys	Laboratory	Decrease in distance flown, flight speed, length of initial flight and length of longest flight	(Schiefer et al., 1977)	Oocysts<br>Sporozoite	
		Anopheles Stephensi	*Plasmodium Yoelii*	Infected Mice	Laboratory	Reduction in flight activity	(Rowland and Boersma, 1988)	Oocysts<br>Sporozoite	
		Aedes Aegypti	*Plasmodium Gallinaceum*	Infected Chicken	Laboratory	Sucked less blood, laid fewer eggs	(Freier and Friedman, 1976)	Sporozoite	

						and showed direct relation between time spent on infected chicken and blood meal size			
		Anopheles Stephensi	*Plasmodium Yoelii Nigeriensis*	Infected Mice	Laboratory	Eggs laid, fecundity and blood meal size reduced	(Hogg and Hurd, 1995b)	Oocyst<br>Sporozoite	
		Anopheles Stephensi	*Plasmodium Yoelii Nigeriensis*	Infected Mice	Laboratory	Fecundity and blood meal size reduced	(Hogg and Hurd, 1995a)	Oocyst	
		Aedes Aegypti (6 different strains based	*Plasmodium Gallinaceum*	Infected White Rock and	Laboratory	Reduced Fecundity	(Hacker, 1971)	Sporozoite	

		on the collection sites)		Leghorn chicks					
		Aedes Aegypti	*Plasmodium Gallinaceum*	Infected White Rock and Leghorn chicks	Laboratory	Reduced fecundity at lower parasite density	(Hacker and Kilama, 1974)	Sporozoite	
		Anopheles Gambiae	*Plasmodium Falciparum*	Human	Field	Reduced fecundity	(Hogg and Hurd, 1997)	Oocyst<br>Sporozoite	
		Anopheles Gambiae	*Plasmodium Yoelii Nigeriensis*	Infected Mice	Laboratory	Reduced fecundity and fertility	(Ahmed et al., 1999)	Oocyst	
		Anopheles Stephensi	Mixture of 2 Genotypes of *Plasmodium Chabaudi*	Infected Mice	Laboratory	Significant reduction in fecundity	(Ferguson et al., 2003)		
		Anopheles Stephensi	*Plasmodium Yoelii Nigeriensis*	Infected mice	Laboratory	Reduction in number of eggs laid	(Hogg and Hurd, 1995b)	Oocyst and Sporozoite	

		Anopheles Stephensi	*Plasmodium Yoelii Nigeriensis*	Infected mice	Laboratory	Reduced fecundity	(Hurd, 1999)	Oocyst and Sporozoite	Reduction was highest during 1st gonotrophic cycle and lowest during 3rd
	Dengue								
		Aedes Aegypti	*DENV-2*	Leibovitz L-15 containing DENV-2	Laboratory	Enhanced locomotor activity	(Lima-Camara et al., 2011)		
		Aedes Aegypti	*DENV-2*	Infected chicken blood	Laboratory	Higher locomotion during night	(Gaburro et al., 2018a)		
		Aedes Aegypti	*DENV-2*	Infected mice	Laboratory	Enhanced host seeking ability	(Tallon et al., 2020)		
		Aedes Aegypti	*DENV-2*	Infected mice	Laboratory	Higher biting rate	(Luz et al., 2011)		

		Aedes Aegypti	*DENV-2*	Infected mice	Laboratory	Longer blood ingestion time	(Sylvestre et al., 2013)		
		Aedes Aegypti	*DENV-3*	Infected mice	Laboratory	Longer feeding time	(Platt et al., 1997)		
		Aedes Aegypti	*DENV-2*	Infected mice	Laboratory	Longer probing and feeding times	(Sim et al., 2012)		
		Aedes Aegypti	*DENV-2*	Infected mice	Laboratory	Decreased motivation to feed and increased avidity	(Maciel-de-Freitas et al., 2013)		
		Aedes Aegypti	*DENV-2*	Infected chicken blood	Laboratory	Olfactory preferences changed	(Gaburro et al., 2018b)		
		Aedes Aegypti	*DENV-2*	Infected mice	Laboratory	Reduction in Fecundity	(Maciel-de-Freitas et al., 2011)		
	Zika								

		Aedes Aegypti		Infected chicken blood	Laboratory	Increased diurnal locomotion activity	(Gaburro et al., 2018a)		
		Aedes Aegypti		Infected mice	Laboratory	Locomotion decreased	(Padilha et al., 2018)		
		Aedes Aegypti		Patient's blood	Laboratory	Fecundity reduced, number of eggs laid per female during the later clutches increased and low but stable egg production per µL of blood ingested	(Petersen et al., 2018)		

		Aedes Aegypti		Rabbit blood	Laboratory	Infertility increased	(Resck et al., 2020)		
	Chikungunya								
		Aedes Aegypti		Rabbit blood	Laboratory	Egg fertility of young and old mosquitoes reduced	(Resck et al., 2020)		
		Aedes Albopictus		Infected Mouse	Laboratory	Shortened the time before oviposition	(Martin et al., 2010)		
	West Nile Virus								
		Culex Tarsalis		Infected Chicken	Laboratory	Lower fecundity during the first oviposition, smaller egg rafts, and	(Styer et al., 2007)		

						lower egg hatch rates			
		Culex Pipiens		Infected Chicken	Laboratory	Host seeking reduced	(Vogels et al., 2017)		
	La Crosse								
		Aedes Triseriatus		Infected Mice	Laboratory	Meal size decreased and avidity increased	(Jackson et al., 2012)		
		Aedes Albopictus		Infected Mice	Laboratory	Meal size decreased and avidity remained constant	(Jackson et al., 2012)		
		Aedes Albopictus		Infected Mouse	Laboratory	Meal size decreased	(Grimstad et al., 1980)		
	Lymphatic								
		Aedes Aegypti	*Brugia Malai*	Infected Human Blood	Laboratory	Flight distance, average flight speed,	(Somerville et al., 2019)	L3 (Developed)	

						and maximum flight speed reduced and flight bursts increased			
		Aedes Aegypti	*Burgia Pahangi*	Infected Cats	Laboratory	Reduced the flight length and overall flight time and increased the number of non flying mosquitoes	(Hockmeyer et al., 1975)		
		Aedes Aegypti	*Burgia Pahangi*	Infected Cats	Laboratory	Nonflying mosquitoes increased with the development of parasite	(Townson, 1970)		

		Aedes Aegypti	*Brugia Malayi*	Infected human blood	Laboratory	Reduced the fecundity	(Gleave et al., 2016)		
Ticks									
	Borrelia								
		Ixodes Ricinus	*Borrelia Burgdorferi*	Collected already infected ticks	Laboratory	Reduced locomotor activity of adults and immatures	(Alekseev et al., 2000)		
		Ixodes Scapularis	*Borrelia Burgdorferi*	Infected Mice	Laboratory	Adults ability to overcome physical obstacles decreased, started avoiding vertical surfaces, less active, and	(Lefcort and Durden, 1996)		

						quested at lower heights.			
		Ixodes Scapularis	*Borrelia Burgdorferi*	Infected Mice	Laboratory	Nymphs questing height increased, greater tendency to overcome physical obstacles, higher phototaxis attraction, and boost in vertical surfaces appeal	(Lefcort and Durden, 1996)		
		Ixodes pacificus	*Borrelia Burgdorferi*		Field	Higher densities of	(Robert et al., 2007)		

						nymphs found on logs and trunks than in the leaf litter			
		Ixodes persulcatus	*Borrelia Burgdorferi*	Collected already infected ticks	Laboratory	Increased attraction of nymphs towards lower temperature areas and adults tolerance to higher temperatures enhanced	(Alekseev and Dubinina, 2000)		
		Ixodes ricinus	*Borrelia Afzelii*	Naturally Infected	Field	Host sensing ability of	(Faulde and Robbins, 2008)		

						adults increased			
	Anaplasma								
		Ixodes scapularis	*Anaplasma Phagocytophilum*	Naturally Infected	Field	Survival of nymphs in cold areas increased	(Neelakanta et al., 2010)		Found more in cold areas compared to uninfected ones
		Ixodes scapularis	*Anaplasma Phagocytophilum*	Nymphs on infected rabbits and adults on infected sheep.	Laboratory	Reduced questing speed	(Busby et al., 2012)		Questing speed reduced by 50% at 4 ºC and 66% at 22 ºC
	Babesia								
		Boophilus Microplus	*Babesia Bovis*	Infected bovines	Laboratory	Significantly reduced the eggs quantity,	(Davey, 1981)		

						eggs masses, and oviposition period			
		Boophilus Microplus	*Babesia Bigemina*	Infected Calves	Laboratory	Reduced the egg production	(De Vos et al., 1989)		
		Boophilus Decoloratus	*Piroplasm Babesia Bigemina*	Infected Calves	Laboratory	Reproduction reduced significantly	(Gray, 1982)		
		Ixodes Trianguliceps	*Babesia Microti*	Infected mice and volves	Laboratory	Increased the feeding success	(Randolph, 1991)		Success not dependent on the infection's density
		Ixodes Scapularis	*Babesia Microti*	Infected Mice	Laboratory	Increased feeding time and engorgement	(Hu et al., 1997)		Host Dependant

	Tick-borne encephalitis virus (TBEV)								
		Ixodes ricinus		Infected Mice	Laboratory	More aggressive and active and are found very often on humans, Tolerant and active against DEET repellent.	(Belova et al., 2012)		After infection, virus replication convert the undetectable concentration of TBEV to a detectable concentration

Table S2: Behaviour monitoring tools to examine mosquito's behaviours

Behaviour		Software/Tool	Computer Software/Smartphone App/ Equipment	Open source/Licensed/ Custom Build/Available for purchase	Auto/Semi Auto	Additional Tools Required	Tracking Dimensions	References
Fitness								
	Flight							
		Mosquito flight mill	Equipment	Custom build	Auto	WinDAQ Lite software, Python	2D	(Au - Attisano et al., 2015)
		Flybox	Equipment	Custom build	Auto		2D	(Araujo et al., 2020)
		Trikineticks activity monitors	Equipment	Available for purchase	Auto	DAMSystem 3 Software	2D	(TriKinetics, 2018)
		Id tracker	Computer Software	Open Source	Auto	Matlab	2D	(Pérez-Escudero et al., 2014)
		Idtracker.ai	Computer Software	Open Source	Auto	Python	2D	(Romero-Ferrero et al., 2019)
		Trackit 3D	Equipment	Available for purchase	Auto	Python	3D	(Hawkes and Gibson, 2016)

		NoldusTrack3D	Computer Software	Available for purchase	Auto		3D	(Noldus, 2020)
	Fecundity							
		Icount	Computer Software	Open Source	Auto & Semi-Auto			(Gaburro et al., 2016)
		Eggcounter 1.0	Computer Software	Open Source	Semi-Auto			(Mollahosseini et al., 2012)
		Mosquito Egg Computer Vision	Web App (Desktop, Smartphone)	Open Source	Semi-Auto			(Krieshok and Torres Gutierrez, 2020)
		AedesTech Apps (ATA)	Smartphone App	Open Source	Auto	MATLAB AutoCountMe System		(Man et al., 2020)
		Macroinstruction	Equipment	Custom Build	Semi-Auto	AxioVision Rel. 4.8 Software		(Dembo et al., 2014)
		Digital Image Analysis Based Method	Equipment	Custom Build	Auto	ImageJ Program		(Mains et al., 2008)
	Reproductive Capacity							
		Mosquito Larvae Counter	Equipment	Custom Build	Semi-Auto			(Mamai et al., 2019)

Blood Feeding								
	Host Seeking							
		Photoelectric Sensing Device	Equipment	Custom Build	Auto		2D	(Kawada and Takagi, 2004)
	Probing and Engorgement							
		BiteOscope	Equipment	Custom Build	Auto	DeepLabCut	2D	(Hol et al., 2020)
